# PSMA PET for primary lymph node staging of intermediate and high-risk prostate cancer: an expedited systematic review

**DOI:** 10.1186/s40644-020-0290-9

**Published:** 2020-01-23

**Authors:** Lars J. Petersen, Helle D. Zacho

**Affiliations:** 1 Department of Nuclear Medicine and Clinical Cancer Research Centre, Aalborg University Hospital, Hobrovej 18-22, DK-9100 Aalborg, Denmark; 2 Department of Clinical Medicine, Aalborg University, Sdr. Skov Vej 15, DK-9000 Aalborg, Denmark

**Keywords:** Prostate cancer, Prostate-specific membrane antigen, Positron emission tomography, Staging, Lymph node metastasis, Diagnostic accuracy

## Abstract

**Background:**

PSMA PET is a promising method for primary lymph node staging in prostate cancer. However, recent systematic reviews have identified only a limited number of studies with histopathology as a reference test.

**Methods:**

A systematic search was performed in PubMed and the Cochrane Library. An expedited systematic review was performed where we identified diagnostic studies in prostate cancer where a preoperative PSMA PET for primary lymph node staging was compared to histopathology. The trials must have diagnostic data on a patient level.

**Results:**

Eighteen eligible clinical trials included 969 patients. The median patient number per study was 32 (range 10 to 208). Five trials were prospective, and nine trials had a consecutive enrolment of patients. Sixteen studies used Ga-68-PSMA-11; there was one study with Cu-64-PSMA and one study with F-18-DCDFPyL. Twelve studies used PET/CT, four trials used PET/MR. Most trials included patients with intermediate and high-risk. Diagnostic accuracy varied notably among the studies; sensitivity ranged from 23 to 100%, specificity 67–100%, positive predictive value 20–100%, and negative predictive value 41–100%. Weighted sensitivity was 59%, weighted specificity was 93%. Four studies compared PSMA PET with anatomical imaging (CT or MRI); in all cases, sensitivity and specificity were superior with PSMA PET. Three studies compared PSMA PET with multi-parametric or diffusion-weighted MRI with mixed results.

**Conclusions:**

PSMA PET showed promising diagnostic accuracy for primary lymph node staging with pathology as reference. Recommendation for PSMA PET for high-risk patients in clinical guidelines should be supported by confirmatory, prospective trials with patient-relevant outcomes.

## Background

Most urological societies recommend aorto-abdominal computed tomography (CT) or magnetic resonance imaging (MRI) for primary lymph node staging of patients with intermediate and high-risk prostate cancer [1–3]. These recommendations have been consistent for many years despite the modest positive and negative predictive values of anatomical imaging for lymph node metastases [4]. Positron emission tomography (PET) tracers like F-18- or C-11-choline have not shown superior to anatomical imaging methods [5].

Within the last couple of years, a high number of publications have indicated an excellent diagnostic performance of prostate-specific membrane antigen (PSMA) PET/CT for prostate cancer, primarily in biochemical recurrence [6–9]. Also, some reports have shown encouraging data with PSMA PET/CT for the detection of lymph node metastases for primary staging. A notable number of systematic reviews have been published on this topic since 2018 [8–13]. These systematic reviews comprise a limited number (five to eight) original reports with primary staging with pathology as reference. We are aware of many more studies with PSMA PET/CT for primary staging than previously reported. Thus, the objective was to provide an overview of trials with PSMA PET for staging of prostate cancer.

## Methods

### Aim

The aim was to provide an up-to-date overview of diagnostic test accuracy trials of preoperative PSMA PET for primary lymph node staging on a patient level in prostate cancer patients with a final pathology reference for lymph node metastasis.

### Study design

This expedited systematic review was mostly compliant with the Preferred Reporting Items for Systematic Reviews and Meta-Analyses (PRISMA) guidelines for reporting of systematic review [14]. The term ‘expedited’ indicates that some aspects of systematic review methodology were kept at a minimum, e.g., one person did the entire eligibility screening, and assessment of bias comprised assessment of study design and recruitment only. Due to the sequential search of papers, a flow diagram of papers was not feasible. The review synopsis for this study was not published in a public database. No synthesis of results (meta-analysis) was performed. The expedited methodology was chosen since the publication velocity of papers within this area is so fast, that a full systematic review would be largely outdated at the time of publication.

### Literature search

A literature search was performed in PubMed, which was searched from inception up to August 16, 2019. We used the following search string: (lymph node metastasis OR staging) AND (positron emission tomography OR PET) AND (prostate specific membrane antigen OR psma) AND (prostate malignancy OR prostatic carcinoma OR prostate cancer OR prostatic neoplasm). We did several searches in PubMed since end-2018 with repetitive and overlapping updates. A final search performed on August 16, 2019, provided 413 references (see Additional file 1). In addition, The Cochrane Library was searched from inception through July 2, 2019, using the search word ‘prostate specific membrane antigen’ or ‘prostate-specific membrane antigen’ (all text search). A total of 123 hits were identified, none of which were relevant supplementary hits compared to the references identified in PubMed. A final search on August 16 also showed 123 references. The validity of the PubMed search string was verified by the identification of all references presented in the latest systematic reviews of 68Ga-PSMA PET/CT for primary staging with pathology as the reference [8–11, 13]. All references were imported into EndNote X8 (Clarivate Analytics, Philadelphia, US) and were evaluated for inclusion in the review. We did not perform additional literature searches (‘grey’ literature, review of reference lists of eligible papers, or contact to authors).

### Eligibility criteria

According to the PICOS concept (patient, intervention, comparator, outcome, study type), the following eligibility criteria were used: 1) The trial must comprise patients diagnosed with prostate cancer; 2) the patients underwent preoperative PSMA PET for primary lymph node staging, and 3) the reference test was histopathology. In addition, 4) the trial must report individual data or summary diagnostic accuracy findings on a patient level; and 5) it should include a minimum of five patients per study fulfilling all the criteria mentioned above. Based on the criteria mentioned above, 6) the study design was limited to diagnostic test accuracy studies. All languages were accepted, and any.

PSMA ligands were eligible. Among papers with mixed types of cancers or settings (e.g., primary and secondary staging), data should be extractable in accordance with the eligibility criteria mentioned above.

### Sorting and data extraction

All papers were initially reviewed for eligibility by the reading of title and abstract by a trained reader (LJP). Apparently ineligible papers were excluded. Potentially eligible papers were reviewed in full text by reading supported by electronic searches within the text for relevant words and phrases, e.g., lymph node, lymph node metastasis, sensitivity, and true positive, and false positive. For papers finally included in the review, two readers (LJP and HDZ) independently extracted the data. A consensus decision was made in the cases of disagreement.

Each paper was reviewed for the reporting of diagnostic performance versus pathology reference (e.g., sensitivity, specificity, positive and negative predictive values) on a patient level. If diagnostic accuracy data were reported by the authors, these figures were used. If data were not reported (or incompletely reported), diagnostic accuracy was determined from 2 × 2 tables of true and false positive and negative cases. Any data available for comparative index tests were also extracted. Some papers reported findings with pathology as the reference in a subset of the patients. In these cases, we restricted the study population to the patients with a pathology reference. If the prostate cancer risk classification was not reported separately for the subset of patients with a pathology reference, the risk class for classified as ‘not reported’. If the study population in a paper comprised patients with primary and secondary lymph node dissection (LND), the mean (or median, if reported) number lymph nodes removed from the entire population was reported if data was not available from patients with primary LND.

### Assessment of bias

Information about patient enrollment (prospective versus retrospective) and selection of patients (consecutive or non-consecutive enrolment) was extracted. The assessment of trial methodology was based on the actual reporting. Thus, a trial was classified as prospective only if the word ‘prospective’ was mentioned, the terminology was clear (e.g., “we enrolled”), or the trial was classified as an interventional trial. Finally, consecutive recruitment of patients was acknowledged only if the phrase “consecutive” or “unselected” was used, or it was clear that the trial included all patients or an unbiased selection of patients examined in a well-defined period, and the eligibility criteria were specified.

### Statistics

Weighted analysis of sensitivity and specificity was performed using the reported diagnostic accuracy data corrected for the number of patients in each trial. No analytical statistics were used.

## Results

### Description of the included papers

The total search strategy comprised 512 references, 389 references from PubMed (Additional file 1) and 123 references from The Cochrane Library. Due to the sequential and overlapping searches, a flow chart cannot be made of the papers read by full text and the reasons for exclusion. The main reason for exclusion of papers after full text reading was the lack of a pathology reference of lymph node metastasis (reporting of detection rates only). One paper selectively reported diagnostic accuracy data on a lesional level with pathology as the reference but no data on the patient level [15].

Eighteen studies, originating from ten countries (based on the country of the first author) and published from 2016 and onwards (PubMed availability), reported data on a patient level (Table 1). Most trials came from Germany (*n* = 4) and Australia (*n* = 3). Most papers used Ga-68-PSMA-11 (PSMA HBED-CC). PET/CT was most frequently used; a few studies used PET/MR (entirely or predominantly). The majority of studies were retrospective in design. Half of the studies recruited consecutive patients; there was only one prospective study with consecutive recruitment. Generally, all papers reported the distribution of patients with intermediate and high-risk or reported proportions of patients with specific T-stages, PSA levels, or Gleason scores. Even though some papers did not report risk classes among patients with pathology as a reference, most of these papers entire or preferable recruited intermediate and high-risk patients [23, 32]. All papers but one performed LND with radical prostatectomy; one study solely included patients who had laparoscopic LND prior to curatively intended radiotherapy [33].

**Table 1:** Study demographics. Data are from patients undergoing staging for newly diagnosed disease in whom a pathology reference was applied

Ref	Author	Year	Country	Tracer	Scanner	Study design	Recruitment	Patients, numbers	Risk group distribution	Mean number of LN removed per patient
1	Budaus et al. [16]	2016	Germany	Ga-68-PSMA-11	PET/CT	Retro	Non-con	30	High (100%)	20
2	Herlemann et al. [17]	2016	Germany	Ga-68-PSMA-11	PET/CT	Retro	Con	20	Int (20%), High (80%)	14
3	Maurer et al. [18]	2016	Germany	Ga-68-PSMA-11	PET/MR (73%), PET/CT (27%)	Retro	Con	130	Int (32%), High (68%)	21^a^
4	Van Leeuwen et al. [19]	2017	Australia	Ga-68-PSMA-11	PET/CT	Pro	Non-con	30	Int (10%), High (90%)	18
5	Uprimny et al. [20]	2017	Austria	Ga-68-PSMA-11	PET/CT	Retro	Non-con	49	NR	NR
6	Gupta et al. [21]	2017	India	Ga-68-PSMA-11	PET/CT	Retro	Non-con	12	High (100%)	20
7	Obek et al. [22]	2017	Turkey	Ga-68-PSMA-11	PET/CT	Retro	Non-con	51	High (100%)	20
8	Zhang et al. [23]	2017	China	Ga-68-PSMA-11	PET/CT	Retro	Con	42	NR	15
9	Park et al. [24]	2018	USA	Ga-68-PSMA-11	PET/MR	Pro	Non-con	33	High (45%), Int (55%)	12
10	Van Leeuwen et al. [25]	2018	The Netherlands	Ga-68-PSMA-11	PET/CT	Retro	Con	140	Int (21%), High (79%)	NR
11	Gorin et al. [26]	2018	USA	F-18-DCFPyL	PET/CT	Pro	Non-con	25	High (100%)	13
12	Cantiello et al. [27]	2018	Italy	Cu-64-PSMA	PET/CT	Pro	Non-con	23	Int (83), High (17)	18
13	Thalgott et al. [28]	2018	Germany	Ga-68-PSMA-11	PET/MR	Retro	Con	73	High (100%)	26
14	Berger et al. [29]	2018	Australia	Ga-68-PSMA-11	PET/CT	Retro	Non-con	50	NR (pT3, 54%; Gleason ≥8; 32%)	12^a^
15	Gupta et al. [30]	2018	India	Ga-68-PSMA-11	PET/CT	Retro	Con	23	NR (83% High based on PSA and T-stage)	20
16	Yaxley et al. [31]	2019	Australia	Ga-68-PSMA-11	PET/CT	Retro	Con	208	High (59%), Int (41%(	14
17	Yilmaz et al. [32]	2019	Turkey	Ga-68-PSMA-11	PET/CT	Retro	Con	10	NR	NR
18	Petersen et al. [33]	2019	Denmark	Ga-68-PSMA-11	PET/MR (85%), PET/CT (15%)	Pro	Con	20	High (95%), Int (5%)	29

^a^Median

Abbreviations. *Con* consecutive, *Int* intermediate, *LN* lymph node, *Non-con* non-consecutive, *NR* not reported, *Pro* prospective, *Retro* retrospective

### Diagnostic accuracy for PSMA PET for lymph node metastasis

Diagnostic accuracy for the detection of lymph node metastasis on a patient basis showed wide variations (Table 2). Sensitivity ranged from 23 to 100%, specificity 67–100%, positive predictive value 20–100%, and negative predictive value 41–100%. However, 15 of 18 studies reported a sensitivity of 50% or greater. Specificity was generally good, 90% or better in most studies. A weighted analysis showed a sensitivity of 59% (specificity 93%) based on the reported diagnostic accuracy data and the number of patients in the respective trials. The disease prevalence varied notably from 4 to 65%. There was some variation of the positive and negative predictive values, but the majority of trials presented values of 80% or better for both positive and negative predictive values.

**Table 2:** Diagnostic performance on a patient basis for the detection of lymph node metastasis with pathology as reference. Data accuracy data from any comparative index test(s) are shown in parenthesis

Ref	Author	LNM (%)	Sens (%)	Spec (%)	PPV (%)	NPV (%)	Comparative index tests
1	Budaus et al. [16]	40	33	100	100	69	None
2	Herlemann et al. [17]	NR	91	67	83	80	None
3	Maurer et al. [18]	32	66 (44)	99 (85)	96 (58)	86 (77)	CT
4	Van Leeuwen et al. [19]	37	64	95	88	82	None
5	Uprimny et al. [20]	37	61	94	85	81	None
6	Gupta et al. [21]	58	100 (57)	80 (80)	88 (80)	100 (57)	MRI
7	Obek et al., [22]	29	53 (25)	86 (76)	61.5 (NR)	81 (NR)	MRI/CT
8	Zhang et al. [23]	36	93 (96)	96 (96)	93 (88)	96 (100)	mpMRI
9	Park et al. [24]	9	100	87	43	100	None
10	Van Leeuwen et al. [25]	36	53 (14)	88 (99)	71 (81)	76 (67)	DW-MRI
11	Gorin et al. [26]	28	71	89	71	89	None
12	Cantiello et al. [27]	35	88	100	100	94	None
13	Thalgott et al. [28]^a^	34	60	100	100	83	None
14	Berger et al. [29]	4	50 (NR)	92 (NR)	20 (NR)	98 (NR)	mpMRI
15	Gupta et al. [30]	39	78	93	88	87	None
16	Yaxley et al. [31]	26	38	93	68	81	None
17	Yilmaz et al. [32]	20	100 (100)	100 (38)	100 (29)	100 (29)	mpMRI
18	Petersen et al. [33]^b^	65	23–39 (8;37)	100 (100;83)	100 (100;80)	41–47 (37;42)	CT/MRI; DW-MRI

^a^Patients with equivocal PSMA scans were consider negative for lymph node metastases. ^b^Sensitivity analysis were performed where intermediate findings are calculated as either benign or malignant). Abbreviations. *LNM* lymph node metastasis

### Comparison of PSMA PET with other index tests

A subset of reports compared PSMA PET with other index tests (Table 2). Four papers compared PSMA PET with guideline-recommended anatomical imaging (CT or MRI). In all four reports, PSMA PET numerically outperformed cross-sectional anatomical imaging. A few studies applied multi-parametric or diffusion-weighted MRI for lymph node staging with heterogeneous findings when compared to PSMA PET.

## Discussion

Cross-sectional anatomical imaging has been recommended for patients with unfavourable intermediate and high-risk prostate cancer for primary lymph node staging across urological guidelines for years despite known insufficient diagnostic accuracy for both CT and MRI [4]. With a weighted sensitivity of 42%, a specificity of 82%, and a positive predictive value of 32%, CT is not the ideal imaging method in this setting. Here we summarised findings from 18 diagnostic test accuracy trials with PSMA PET and a valid reference test and found a weighted sensitivity of 59%, a specificity of 93%, and positive and negative predictive values of 80% or higher in the majority of the trials. These findings indicate that PSMA PET should be considered for primary staging in prostate cancer.

PSMA PET has shown encouraging diagnostic properties in prostate cancer since the first clinical trial was published with Ga-68-PSMA-11 in 2012 [34]. Since then, the use of PSMA PET has been evaluated in numerous settings, including primary staging and biochemical recurrence, as shown in recent systematic reviews [8–13]. However, these recent systematic reviews only present a fraction of the knowledge of PSMA PET for primary lymph node staging. Even though our systematic literature search included all PSMA ligands, 16 of 18 trials were with Ga-68-PSMA. The clinical trial documentation with PSMA PET originated from 2016 and onwards with most trials published in 2018 and later. It may be speculated that the tremendous work with systematic reviews and meta-analysis, including duplicate search and selection, searched across several databases and the grey literature, detailed bias assessment, may impair the ability to show up-to-date evidence. With rapidly evolving methods like PSMA PET, expedited systematic literature reviews may better provide a status of the current knowledge. We certainly acknowledge the place for systematic reviews and meta-analyses in particular to synthesise cumulative evidence in other areas of medicine.

The 18 trials showed clinical compelling diagnostic performance in those patients where primary lymph node staging is indicated (high-risk and unfavourable intermediate-risk categories), even though some variations in diagnostic accuracy were observed across the trials. The reasons for the wide variations may, among others, be related to research design, technical aspects of the PET/CT scanner and image reconstruction, reader experience, the surgical procedure, and handling of the pathology specimens. Most studies were of modest size, which together with variations in the proportion of LN metastatic patients (4 to 65%) may influence precision of the diagnostic test characteristics. Also, it has been documented that wide disease spectrum, non-consecutive recruitment, open-label reading of tests, and retrospective data collection were associated with higher estimate of diagnostic accuracy [35]. In this review [33, 36], the proportion of high-risk patients varied from 17 to 100%, approximately 50% of the trials stated consecutive recruitment, and blinding of readers were seldom reported. There were five prospective trials, but only one prospective, interventional trial with consecutive enrollment [33]. That study was fully compliant with The Standards for Reporting of Diagnostic Accuracy (STARD) [37], performed according to the Good Clinical Practice requirements, but unfortunately performed with older PET/CT scanners. Reader experience with PSMA PET should also be taken into consideration [38]; reader experience was seldom mentioned in the 18 trials. In addition to the extent of surgical resection, details for pathology assessment, including criteria for a pathology-positive lymph node, should be considered; such details were infrequently reported.

The weighted sensitivity and specificity of PSMA PET outperformed the reported diagnostic accuracy of CT and MRI published in the latest meta-analysis [4]. Four trials directly compared PSMA PET with cross-sectional imaging. In all trials, PSMA PET numerically outperformed CT and MRI. A few trials compared PSMA PET with functional MRI; the findings were heterogeneous, so no firm conclusion could be made.

The differences in diagnostic accuracy among anatomical and functional imaging methods may mainly reflect the criteria for classifying an index test as positive for malignancy. With CT and MRI, a size criteria of 10 or 15 mm, with or without additional radiological criteria, are generally applied for pathological lymph nodes [4, 39]. PET criteria combine anatomical and functional findings with quite consistent observer agreement across reporting guidelines [40]. Equivocal imaging findings on PSMA (e.g. PSMA-RADS-3) still represent a challenge; however, most studies in this systematic review reported PSMA results on a dichotomous scale. Handling of equivocal findings can notable influence diagnostic accuracy [41]. Recent studies with PSMA PET have revealed that most lymph node metastases are in fact quite small. In our own recent trial, false-negative metastases on PSMA PET/CT had a median diameter of 4 mm, with only 3 of 30 lymph nodes being larger than 10 mm; 31% of the patients with lymph node metastases had micrometastasis (< 0.2 mm) only [33].

When we searched for eligible papers for this review, it was striking that approximately 50% of the papers published in this area reported detection rates only, not correlative findings with pathology. At staging, it is possible to do pathological staging, at least on a patient basis. In other settings, e.g. biochemical recurrence, pathology may be difficult to obtain. Such results should be carefully evaluated due to possible verification bias [42, 43] [33, 37]. In order to move PSMA PET higher in the hierarchy of clinical evidence, we not only need large prospective diagnostic test accuracy trials [44], but we also need trials showing improvement of patient benefit, e.g. improved outcome of having a preoperative PSMA PET rather than CT or MRI. There are indications that such a benefit can be documented; in a recent study by van Leeuwen et al. [25], the biochemical recurrence rate after radical prostatectomy was 17% among patients who had no signs of lymph node metastasis on the PSMA PET/CT compared to 50% of those in whom PSMA identified lymph node involvement. Once clinical benefit with PSMA PET has been documented, strong recommendations in clinical practice guidelines will follow.

## Conclusion

The current level of evidence indicated interesting diagnostic performance of PSMA PET/CT for primary lymph node staging of high-risk prostate cancer patients though there were some variations across the studies. PSMA PET/CT showed superior diagnostic performance compared to anatomical imaging for lymph node staging in studies where comparative data were reported. Since studies have shown PSMA PET/CT to outperform bone scans, with or without SPECT/CT, for the detection of skeletal metastases [45–48], PSMA PET/CT may turn out to be a one-stop modality for staging in these patients.

## Supplementary information

**Additional file 1.** PubMed search (text file)

## Data Availability

The datasets used and/or analysed during the current study are available from the tables, and the references cited.

## Abbreviations

CT: Computer tomography
MRI: Magnetic resonance imaging
PET: Positron emission tomography
PICOS: Patient, intervention, comparator, outcome, and study type
PRISMA: The Preferred Reporting Items for Systematic Reviews and Meta-Analyses
PSMA: Prostate-specific membrane antigen
